# Molecular detection of vector-borne pathogens in blood and splenic samples from dogs with splenic disease

**DOI:** 10.1186/s13071-017-2074-z

**Published:** 2017-03-13

**Authors:** Rebeca Movilla, Laura Altet, Lorena Serrano, María-Dolores Tabar, Xavier Roura

**Affiliations:** 1 Hospital Clínic Veterinari, Universitat Autònoma de Barcelona, Carrer de L’Hospital s/n, 08193 Bellaterra, Cerdanyola del Vallès, Barcelona, Spain; 2 Vetgenomics, Edifici Eureka, Parc de Recerca de la Universitat Autònoma de Barcelona, 08193 Bellaterra, Cerdanyola del Vallès, Barcelona, Spain; 3 Hospital Veterinario San Vicente, Calle del Veterinario Manuel Isidro Rodríguez García N°17, 03690 San Vicente del Raspeig, Alicante, Spain

**Keywords:** Canine, Vector-borne disease, Spleen, Polymerase chain reaction, Hemangiosarcoma, *Babesia canis*

## Abstract

**Background:**

The spleen is a highly perfused organ involved in the immunological control and elimination of vector-borne pathogens (VBP), which could have a fundamental role in the pathogenesis of splenic disease. This study aimed to evaluate certain VBP in samples from dogs with splenic lesions.

**Methods:**

Seventy-seven EDTA-blood and 64 splenic tissue samples were collected from 78 dogs with splenic disease in a Mediterranean area. *Babesia* spp., *Bartonella* spp., *Ehrlichia/Anaplasma* spp., *Hepatozoon canis*, *Leishmania infantum*, hemotropic *Mycoplasma* spp. and *Rickettsia* spp. were targeted using PCR assays. Sixty EDTA-blood samples from dogs without evidence of splenic lesions were included as a control group.

**Results:**

More than half (51.56%) of the biopsies (33/64) were consistent with benign lesions and 48.43% (31/64) with malignancy, mostly hemangiosarcoma (25/31). PCR yielded positive results in 13 dogs with spleen alterations (16.67%), for *Babesia canis* (*n* = 3), *Babesia gibsoni* (*n* = 2), hemotropic *Mycoplasma* spp. (*n* = 2), *Rickettsia massiliae* (*n* = 1) and “Babesia vulpes” (*n* = 1), in blood; and for *B. canis*, *B. gibsoni*, *Ehrlichia canis* and *L. infantum* (*n* = 1 each), in spleen. Two control dogs (3.3%) were positive for *B. gibsoni* and *H. canis* (*n* = 1 each). Benign lesions were detected in the 61.54% of infected dogs (8/13); the remaining 38.46% were diagnosed with malignancies (5/13). Infection was significantly associated to the presence of splenic disease (*P* = 0.013). There was no difference in the prevalence of infection between dogs with benign and malignant splenic lesions (*P* = 0.69); however *B. canis* was more prevalent in dogs with hemangiosarcoma (*P* = 0.006).

**Conclusions:**

VBP infection could be involved in the pathogenesis of splenic disease. The immunological role of the spleen could predispose to alterations of this organ in infected dogs. Interestingly, all dogs with *B. canis* infection were diagnosed with hemangiosarcoma in the present survey. As previously reported, results support that VBP diagnosis could be improved by analysis of samples from different tissues. The sample size included here warrants further investigation.

**Electronic supplementary material:**

The online version of this article (doi:10.1186/s13071-017-2074-z) contains supplementary material, which is available to authorized users.

## Background

Vector-borne pathogens (VBP) are arthropod-transmitted agents, mainly parasites and bacteria, which cause a significant impact on the health of dogs. In addition to their veterinary importance, dogs can act as reservoirs for VBP of major zoonotic concern [1]. The risks of canine vector-borne diseases (CVBD) infection and the severity of clinical signs are a consequence of a complex interrelationship between the infectious agent, the arthropod vector and immune response of the dog [2]. Substantial evidence supports that the spleen plays a key role in the immunopathology of CVBD [3]. However, the development of splenic disease in itself has also been anecdotally associated with the presence of VBP infections in dogs [4, 5].

The spleen is a secondary lymphoid organ specialized in filtering blood-borne pathogens and antigens by both innate and adaptive immune response. The splenic red pulp contains macrophages that trap and remove damaged red blood cells. The blood flow pattern and a subset of specialized macrophages ensure that the antigen-presenting cells capture and deliver these blood-borne antigens to the B cells in the marginal zone. Afterwards, antibody-producing cells migrate from the primary follicles in the white pulp, colonize the marginal zone and move into the red pulp, where antibody production occurs before blood returning to the circulation [6]. Further characterization of the immunological phenomena, developed in the spleen of VBP-infected dogs and other mammals, remains subject of continuous investigation. The acute splenic response of calves to *Babesia bovis* was remarkably similar to that reported in a mouse model of malarial infection indicating that dynamic changes in the distribution of splenic cells is common in the acute response to the hemoparasitic infection [7]. It was shown that the spleen is able to retain *Bartonella* spp. using an experimental mouse model, although the mechanisms of retention have still to be elucidated [8]. During the acute phase of experimentally induced canine monocytic ehrlichiosis, high levels of tumor necrosis factor (TNF)-α expression were found in splenocytes, CD8+ cells were decreased while CD3+ cells were increased, which could have occurred as an immunologic modulation in the spleen contributing to the pathogenesis. Furthermore, immunohistochemistry revealed a higher number of immunoglobulin (Ig) M and IgG immunolabeled cells responsible, at least in part, for the morphological changes observed in this organ [9, 10].

Several reports have been focused on describing the cellular immunophenotypic profile in the splenic compartment and the co-expression of Th1 pro-inflammatory cytokines [such as interferon (IFN)-γ, interleukin (IL)-12 and TNF-α] and Th2 anti-inflammatory/regulatory cytokines [such as tumor growth factor (TGF)-β, IL-4 and IL-10], throughout the course of natural and experimental visceral leishmaniosis [11, 12]. The large number of cells (lymphocytes) undergoing apoptosis in the spleen of dogs with visceral leishmaniosis was correlated with the parasite load, which suggested that this process could be a contributing factor for the survival of the protozoon in this organ [13]. Of note, splenectomy was considered one of the main clinical outbreaks of hemotropic *Mycoplasma* spp. infection in splenectomized dogs [14]. *Theileria equi* was found in the spleen of asymptomatic horses that did not show parasitemia, which suggested that this might be an important tissue supporting the persistence of the parasite in the host [15].

On the other hand, VBP infection can induce changes in the architecture of the spleen. Conditions such as babesiosis that increase the size of this organ elevate the risk of parenchymal disruption [16]. *Bartonella* spp. infection has been associated to the presence of granulomatous splenic lesions, abscess, and vasoproliferative tumors, such as hemangiopericytoma and hemangioendothelioma in dog and human patients, respectively [17–19]. Molecular confirmation supported higher prevalence for *Bartonella* spp. compared to *Babesia* spp. and hemotropic *Mycoplasma* spp. in dogs with fibrohistiocytic nodules (FHN) and hemangiosarcoma (HSA) in the spleen [4]. Mechanistic evidence as to how these bacteria contribute to the development of vasoproliferative lesions has been provided [5]. Additionally, splenic vasculitis, thrombosis and infarction, were recently documented in a febrile dog diagnosed with *Bartonella henselae* [20]. Moreover, a comparative experimental infection study in dogs infected with *Ehrlichia* spp. and *Anaplasma* spp*.* described infiltration of perivascular mononuclear cells into the spleen, more severe for ehrlichial infections [21]. *Leishmania infantum* and *Hepatozoon canis* were identified in the spleen of dogs with splenitis [22]; and progressive spleen architecture breakage in visceral leishmaniosis was characterized by disorganization of the white pulp, associated with a more frequent and intense plasma cell accumulation in this organ, mostly in dogs with high parasite density [23].

The present study aimed to evaluate the prevalence of certain VBP commonly found in the Mediterranean area, such as *Babesia* spp., *Bartonella* spp*., Ehrlichia/Anaplasma* spp., *H. canis*, *L. infantum*, hemotropic *Mycoplasma* spp. and *Rickettsia* spp., in dogs with splenic disease and to investigate potential associations of these pathogens with the development of splenic lesions.

## Methods

### Sample collection

Fresh splenic tissue and/or ethylene diamine tetraacetic acid (EDTA)-blood samples from dogs undergoing splenectomy in two Referral Veterinary Hospitals located in a Mediterranean area, were collected and stored immediately after surgery (-20 °C), between January 2010 and June 2016. Diagnosis of splenic disease was based on a combination of clinical signs, laboratory testing, diagnostic imaging assessment, findings on surgical exploration of the abdominal cavity, and cytological and/or histopathological evaluation of the spleen. For 17 cases in which fresh splenic tissue samples were not collected, available paraffin-embedded surgical biopsy samples were retrieved from pathology archive storage facilities, using the Pathology Data Base. Although not at the same time, splenic tissue samples were processed and paraffin-embedded with the same equipment and techniques. Board-certified veterinary pathologists performed the histopathological evaluation of the splenic tissue samples.

Control EDTA-blood samples were prospectively collected and stored (-20 °C) from dogs with or without clinical signs attending the hospitals for unrelated reasons during the same period, but whose spleens were deemed within normal limits on imaging assessment (ultrasound and/or computed tomography). All splenic tissue and EDTA-blood samples were identified and processed for polymerase chain reaction (PCR).

Signalment was recorded and medical information was reviewed for every dog. Verbal owner consent was received for all dogs prior to their enrolment in the study. This study was carried out in accordance with the International Guiding Principles for Biomedical Research Involving Animals, issued by the Council for the International Organizations of Medical Sciences.

### PCR testing

DNA from splenic tissue was extracted using the Purelink® Genomic DNA Kit following manufacturer instructions (Thermo Fisher Scientific, Waltham, MA, USA). DNA was extracted from 400 μl of whole blood as previously described [24]. Collected samples, previously defined as specific pathogen-free (SPF) by PCR, were used as an extraction negative control in each extraction batch. Samples were tested in an operator blind manner. *Babesia* spp., *Bartonella* spp*., Ehrlichia/Anaplasma* spp., *H. canis*, hemotropic *Mycoplasma* spp. and *Rickettsia* spp. were targeted using specific PCR assays (Table 1). Real time PCR was carried out in a total volume of 20 μl containing SYBR® Select master mix (Thermo Fisher Scientific), specific primer (see Table 1 for sequence and concentration) and 4 μl of 1/5 diluted DNA. The thermal cycling profile was 50 °C for 2 min and 95 °C for 10 min followed by 40 cycles at 95 °C for 15 s and 60 °C for 1 min and a dissociation curve added at the end of the run. Water was used as PCR negative control and commercial DNAs as positive PCR control. In all cases in which splenic tissue and EDTA-blood samples were PCR-positive, direct DNA sequencing was performed to characterize pathogens at the species level. Sequencing was done by the BigDye® Terminator v3.1 Cycle Sequencing Kit (Thermo Fisher Scientific) following the manufacturer instructions and with the same primers used in the PCR and sequences compared with the GenBank database (https://blast.ncbi.nlm.nih.gov/Blast.cgi). The eukaryotic 18S ribosomal ribonucleic acid (rRNA) (Thermo Fisher Scientific) was used as an endogenous control to ensure proper DNA extraction. Quantitative *L. infantum* PCR was performed as described by Francino and collaborators [24].

**Table 1:** PCR targets and primers used in this study for detection of *Babesia* spp., *Bartonella* spp., *Ehrlichia/Anaplasma* spp., *H. canis*, hemotropic *Mycoplasma* spp. and *Rickettsia* spp*.*

Species	Target gene	Forward primer (5′–3′)	Reverse primer (5′–3′)	Final primer concentration (μM)	Reference
*Babesia* spp*.*	18S rRNA	GACGATCAGATACCGTCGTAGTCC	CAGAACCCAAAGACTTTGATTTCTCTC	0.3	[62]
*Bartonella* spp.	ITS1	AGATGATGATCCCAAGCCTTCTG	CCTCCGACCTCACGCTTATCA	0.3	Modified from [63, 64]
*Ehrlichia* spp.; *Anaplasma* spp.	16S rRNA	GCAAGCYTAACACATGCAAGTCG	GGATTATACAGTATTACCCAYCATTTCTARTG	0.5	[65, 66]
*H. canis*	18S rRNA	CTTACCGTGGCAGTGACGGT	ATTGTTATTTCTTGTTACTACCTCTCTCAAAC	0.3	[66]
Hemotropic *Mycoplasma* spp.	16S rRNA	ATGTTGCTTAATTCGATAATACACGAAA	ACRGGATTACTAGTGATTCCAACTTCAA	0.3/0.5	[65]
*Rickettsia* spp.	ITS2	GCTCGATTGRTTTACTTTGCTGTGAG	CATGCTATAACCACCAAGCTAGCAATAC	0.5/0.3	[62]

*Abbreviations*: *rRNA* ribosomal ribonucleic acid, *ITS* internal transcribed spacer

### Statistical analysis

Statistical analysis was performed using SPSS® 20.0 software. Values for the prevalence of *Babesia* spp., *Bartonella* spp*., Ehrlichia/Anaplasma* spp., *H. canis*, *L. infantum*, hemotropic *Mycoplasma* spp. and *Rickettsia* spp. were established. Chi-square and Fisher’s exact test were used to compare proportions of positivity. The level of statistical significance considered was *P* < 0.05.

## Results

A total of 138 dogs were enrolled in this study. Additional file 1: Table S1 shows in detail the signalment, samples collected, histopathological diagnosis and PCR results.

Seventy-eight dogs were included in the group with spleen alterations, 40 female (51.94%) and 37 male (48.05%). Twenty-seven different breeds were represented including crossbreed dogs (*n* = 15), German Shepherd (*n* = 13), Boxer (*n* = 6), English Cocker Spaniel (*n* = 5), Golden Retriever (*n* = 4), Beagle (*n* = 3), French Bulldog (*n* = 3), Great Dane (*n* = 3), Labrador Retriever (*n* = 3), and others (*n* = 22). Age of dogs in this group ranged from 3 to 16 years, with a mean age of 9 years. Signalment was partially unknown for four dogs. The control group included 60 dogs, 35 female (58.3%) and 25 male (41.7%). Twenty-four different breeds were represented including: crossbreed dogs (*n* = 14), Yorkshire Terrier (*n* = 9), Golden Retriever (*n* = 5), Boxer (*n* = 3), German Shepherd (*n* = 3), Miniature Schnauzer (*n* = 3), and others (*n* = 23). Age of dogs in this group ranged from 1 to 16 years, with a mean age of 8.3 years. Histopathology of the spleen was performed in 64 out of 78 dogs with spleen alterations. Benign lesions were observed in the 51.56% (33/64). The most common were lymphoid nodular hyperplasia (LNH) (*n* = 10), congestion (*n* = 9), necrosis (*n* = 7), hematoma (*n* = 5), extramedullary hematopoiesis (*n* = 4), and hemosiderosis (*n* = 3). Some dogs were histopathologically diagnosed with more than one benign splenic lesion. Malignant lesions were observed in the remaining 48.43% (31/64). The most common was HSA (*n* = 25), followed by carcinoma (*n* = 2), lymphoma (*n* = 2), FHN (*n* = 1), and undifferentiated sarcoma (*n* = 1). Nine out of the 78 dogs with spleen alterations were diagnosed with splenic torsion, two with splenic rupture due to a blunt abdominal trauma, and three were euthanized. The owners refused further investigation in these 14 cases, so histopathological assessment of the spleen could not be performed.

PCR was positive for 15 out of 138 dogs included (10.9%). For dogs with spleen alterations, 13 out of 78 (16.67%) were PCR-positive for at least one VBP when either splenic tissue (*n* = 4) or blood (*n* = 9) was tested. On the other hand, only 2 out of 60 control dogs (3.3%) were found PCR-positive for VBP. In the 13 positive dogs, agreement was not found for PCR outcome between splenic tissue and EDTA-blood samples obtained from the same dog. PCR results, type of biological samples analyzed in positive cases, and histopathological diagnosis of the spleen when it was available, are shown in Table 2. Statistical analysis revealed that VBP infection was significantly associated to the presence of splenic disease (*χ* ^2^ = 6.223, *df* = 1, *P* < 0.05). Relationship between VBP infection and the character of the splenic lesions was clinically presumed (*χ* ^2^ = 0.161, *df* = 1, *P* > 0.05). Indeed, all dogs infected with *B. canis* were diagnosed with HSA (*χ* ^2^ = 7.473, *df* = 1, *P* < 0.05).

**Table 2:** Relationship between histopathological examination of spleen tissue samples and vector-borne pathogens detected

Dog	PCR result	Sample	Hystopathology
S1	*Babesia canis*	Frozen spleen	HSA
S2	*Babesia canis*	EDTA-blood	HSA
S3	*Babesia canis*	EDTA-blood	HSA
S4	*Babesia canis*	EDTA-blood	HSA
S5	*Babesia gibsoni*	Paraffin-fixed spleen	EH, hemosiderosis, LNH
S6	*Babesia gibsoni*	EDTA-blood	Congestion, hematoma
S7	*Babesia gibsoni*	EDTA-blood	Congestion
C1	*Babesia gibsoni*	EDTA-blood	na
S8	“Babesia vulpes”	EDTA-blood	na (splenic torsion)
S9	*Ehrlichia canis*	Frozen spleen	Congestion, hematoma
C2	*Hepatozoon canis*	EDTA-blood	na
S10	*Leishmania infantum*	Frozen spleen	EH, myeloid metaplasia
S11	“*Candidatus* Mycoplasma hemovis”	EDTA-blood	Congestion, EH, hemosiderosis
S12	*Mycoplasma ovis*	EDTA-blood	Carcinoma
S13	*Rickettsia massilliae*	EDTA-blood	LNH

*Abbreviations*: *S*, dogs with spleen alterations, *C*, control dogs, *EH* extramedullary hematopoiesis, *HSA* hemangiosarcoma, *LNH* lymphoid nodular hyperplasia, *na* not available

## Discussion

In the study presented here, PCR detected a higher prevalence of VBP in dogs with splenic alterations (16.67%) as compared with dogs without evidence of splenic changes (3.3%), which supports a potential relationship between the pathogenesis of CVBD and splenic diseases.

To the best of our knowledge, the literature contains only one report by Varanat and collaborators on molecular prevalence of certain VBP in dogs with specific splenic conditions [4]. In the latter study, *Bartonella* spp. DNA was more frequently detected in FHN (29.7%), HSA (26%) and LNH (10%) compared to *Babesia* spp*.* (2.7, 2.0 and 2.0%, respectively) and hemotropic *Mycoplasma* spp*.* (0, 6 and 0%, respectively), while all of the paraffin-embedded splenic tissues from dogs with histologically unremarkable spleens were PCR negative [4]. Differences observed between dogs with splenic disease and control dogs in the study of Vranat et al. [4] are in agreement with results obtained here. In contrast, specific prevalence of infection with *Bartonella* spp*.*, *Babesia* spp*.* and *Mycoplasma* spp*.* disagree greatly. Differences in epidemiological conditions and populations tested could explain such discrepancy. However, the absence of *Bartonella* spp*.* DNA in dogs with splenic disease, mainly vasoproliferative tumor-like lesions, was unexpected in our investigation. Molecular evidence supports that four species of *Bartonella*, alone or in co-infection, could potentially induce vasoproliferative lesions [5]. *Bartonella* infection induces overlapping mechanisms in the development of vasoproliferative tumors, such as direct mitogenic effect on endothelial cells, inhibition of endothelial cell apoptosis and activation of hypoxia inducible factor-Iα, resulting in increased expression of the vascular endothelial growth factor [25–28]. In Spain, serological evidence of *Bartonella* spp*.* exposure and molecular detection of the genera have been reported in dogs [29–31]. However, diagnosis of bartonellosis represents a challenge [31] and could be the main cause of the difference in the prevalence of *Bartonella* spp. between the study by Varanat et al. [4] and the present study. The use of a combined approach of serology, pre-enrichment culture of *Bartonella* spp*.* in *Bartonella* alpha proteobacteria growth medium (BAPGM) and PCR may enhance the diagnostic confirmation of bartonellosis in blood or tissue samples of naturally infected dogs [20, 31, 32].

In contrast, *Babesia* spp*.* showed the highest prevalence in the present survey (6.52%) with nine out of 138 dogs infected. Similar rate of molecular detection for *Babesia* spp*.* in EDTA-blood samples (5%) was reported for dogs from the same area presenting for compatible clinical signs and/or criteria with ehrlichiosis during 2011 [33]. The spleen protects against *Babesia* spp*.* by ingestion and clearance of infected red blood cells [34]. Infected erythrocytes could alter spleen histopathology, cause cell cycle alteration (G0/G1, S, and G2/M phases) and induce oxidative stress in splenic tissue, as indicated by decreased glutathione, catalase and superoxide dismutase levels [35]. Damaged red blood cells, iron, free radicals and other reactive oxygen species from macrophage activation produce DNA damage in endothelial cells [36]. Moreover, once activated, macrophages are the main source of growth factors and cytokines (such as TNF-α, IL-12 and IFN-γ), which profoundly affect endothelial, epithelial and mesenchymal cells in the local microenvironment [37–39]. It is now becoming clear that the tumor microenvironment, which is largely coordinated by inflammatory cells, is an essential contributor in the neoplastic process, promoting proliferation, survival and migration [39]. These phenomena coupled together could provide the events necessary for the induction of HSA [36]. In the present study, PCR testing identified four dogs infected with *B. canis* and, interestingly, all of them were diagnosed with HSA. On the other hand, a potential causative role in the development of splenitis has been attributed to the presence of mesenchymal neoplastic diseases [22]. As previously described, HSA could predispose to alter the splenic vasculature, thrombosis, and abnormal blood flows, possibly favoring bacterial engraftment [40].

Hemotropic *Mycoplasma ovis* was PCR-detected in one out of two dogs diagnosed with splenic carcinoma. Many studies have indicated that persistent exposure to *Mycoplasma* spp*.* is closely associated with oncogenic transformation in human cancers and have found that the *Mycoplasma* p37 protein alone is sufficient to increase the invasiveness and metastases of cancer cells [41–44]. *Mycoplasma* spp*.* DNA was successfully extracted and amplified from multiple forms of human carcinoma [44–46]. Experimental data indicated that some *Mycoplasma* spp. cause chromosomal changes and cell transformations in vitro through progressive chromosomal loss and translocations. However, the relationship between *Mycoplasma* spp. infection and cancer progression is yet to be disclosed [45].

Evidence supports that infiltration and activation of inflammatory cells confer favorable conditions for the progression of cancer, inducing angiogenesis, tumor growth and invasion [46]. To elucidate whether VBP induce such infiltration and activation of inflammatory cells in the spleen or alternatively are attracted to pre-existing neoplastic splenic inflammatory lesions, thereby explaining DNA detection of VBP in these dogs, warrants further investigation.

Remaining VBP infected dogs showed benign splenic diseases or were control dogs. Four of these were infected with *B. gibsoni.* In contrast to reported data, breed predisposition was not observed for this infection [47]. Histopathology was not available for the only “B. vulpes”-infected dog that was diagnosed with splenic torsion associated with gastric-dilation volvulus (GDV). Evidence supports that “B. vulpes” is genetically related to *B. gibsoni* [48]. Trotta et al. [49] described a *B. gibsoni-*infected dog with suspected splenic torsion. In such case, histopathological evaluation of the spleen revealed white and red pulp hyperplasia, associated with a diffuse granulocytic infiltrate, hemorrhages and massive necrosis with vascular thrombosis, suggestive of splenic infarct [49]. In addition, a dog with splenic torsion and PCR positive for *L. infantum* was also reported [22]. In this case, the authors considered that both conditions might have triggered splenitis because of altered vascularity and blood supply or activation of the reticuloendothelial system. The etiology of primary splenic torsion (PST) and the association between PST and GDV is unclear, with anecdotal evidence suggesting that dogs with history of PST have an increased risk of developing GDV [50]. Based on the former information, we hypothesize that in the dog of the present study splenic changes associated with VBP infection could have induced PST, and potentially secondary GDV.

*Anaplasma* spp*.* was not detected in any dog. Furthermore, in the present study, prevalence of *Ehrlichia canis*, *L. infantum*, *H. canis* and *Rickettsia massilliae* was lower than expected given previous reports conducted in the same area [29, 30]. These findings combined, suggest that these pathogens may be underestimated in the current study. As previously described, several factors could prompt failure to reach PCR diagnosis, such as small quantity of tissue placed in paraffin blocks from markedly enlarged spleens and the limited quantity of extracted DNA (host and pathogen) that can be incorporated into each PCR reaction, storage conditions and varying periods of formalin fixation that could induce DNA degradation, and/or decreased microorganism load in systemic circulation, possibly associated with the use of specific treatment before obtaining the samples [4, 31, 51].

Serological and PCR assays used in parallel could have maximized CVBD diagnosis [52]. However, in agreement with previous publications, the results obtained in blood and tissue samples from dogs with spleen alterations support that sampling from different tissues improved detection of VBP infection [53–56]. The tropism of certain VBP, such as *L. infantum* caused by lymphoid organs makes them important for detecting this protozoan [56]. It has been shown that positivity increases with disease progression and dogs without clinical signs present lower tissue parasitism, while dogs with clinical signs have high parasite loads in tissues such as the spleen [57]. Moreover, parasites do not distribute themselves evenly in all tissues [58]. Several parameters should be considered in choosing the sample to be used with special care to avoid excessive invasiveness and potential risks for the dog, such as pain, infection or hemorrhage [59].

None of the PCR-positive dogs in the present study showed VBP infection on histopathology of the spleen. As previously described, it is possible that some splenic macrophages had phagocytosed DNA fragments, but intact microorganisms were not detectable, so the amplification methods used with PCR allowed positive results with very small amounts of DNA, while this was not achievable by means of histopathology [22].

There were some limitations in the present study, which have still to be mentioned. Due to the partially retrospective nature of this research and the fact that dogs attended at different institutions, information gathered from available medical records was sometimes incomplete. Furthermore, obtaining optimal age and sex-matched groups was not entirely possible because splenectomy is performed only when there is overt evidence of splenic alterations and splenic biopsies are infrequently obtained. In addition, it is not possible to rule out differences in the processing of the paraffin-embedded tissue samples, retrieved from the pathology archive storage facilities, which were obtained before the frozen-samples collection period. Moreover, control dogs without evidence of splenic alterations were selected based on diagnostic imaging assessment; although ultrasonography and/or radiological splenic changes have shown a good correlation with other laboratory examinations in CVBD, histopathology could have been useful to further confirm the absence of splenic disease in such cases [60, 61]. In addition, samples were stored and retrieved from archives, so prior contamination could not be completely excluded.

## Conclusions

This study provides additional evidence that CVBD could be involved in the pathogenesis of splenic alterations. The high prevalence of *B. canis* infection in dogs with splenic HSA suggests a potential role for this pathogen in the development of vasoproliferative tumors. Furthermore, based on the results obtained here, the presence of splenic disease should prompt further investigation for detection of VBP infection, mainly in endemic areas. Detection of VBP infection is improved by sampling of different tissues.

## Additional file

Additional file 1: Table S1. Details for signalment, collected samples, histopathological diagnosis and PCR results for dogs included in this study. (XLSX 54 kb)

## Abbreviations

BAPGM: *Bartonella* alpha proteobacteria growth medium
C: control dog
CVBD: canine vector-borne disease
DNA: deoxyribonucleic acid
EDTA: ethylene diamine tetraacetic acid
EH: extramedullary hematopoiesis
FHN: fibrohistiocytic nodules
GDV: gastric dilation volvulus
HSA: hemangiosarcoma
IBD: inflammatory bowel disease
Ig: immunoglobulin
IL: interleukin
ITS: internal transcribed spacer
LNH: lymphoid nodular hyperplasia
PST: primary splenic torsion
rRNA: ribosomal ribonucleic acid
S: splenectomized dog
SPF: specific pathogen free
TGF: tumor growth factor
TNF: tumor necrosis factor
VBP: vector-borne pathogens
